# Functional and radiographic outcomes of hallux valgus correction by mini-invasive surgery with Reverdin-Isham and Akin percutaneous osteotomies: a longitudinal prospective study with a 48-month follow-up

**DOI:** 10.1186/s13018-016-0491-x

**Published:** 2016-12-05

**Authors:** Carlo Biz, Michele Fosser, Miki Dalmau-Pastor, Marco Corradin, Maria Grazia Rodà, Roberto Aldegheri, Pietro Ruggieri

**Affiliations:** 1 Orthopaedic Clinic, Department of Surgery, Oncology and Gastroenterology DiSCOG, University of Padua, via Giustiniani 2, 35128 Padova, Italy; 2 Human Anatomy and Embryology Unit, Experimental Pathology and Therapeutics Department, University of Barcelona, Barcelona, Spain; 3 Health Sciences Faculty of Manresa, University of Vic-Central University of Catalunya, Barcelona, Spain; 4 Orthopaedic and Trauma Unit, Padua Hospital, via Giustiniani 2, Padova, Italy

**Keywords:** Hallux valgus, Reverdin-Isham osteotomy, Akin osteotomy, Minimally invasive surgery, Percutaneous distal osteotomy, First ray, Forefoot

## Abstract

**Background:**

Minimally invasive surgery (MIS) represents one of the most innovative surgical treatments of hallux valgus (HV). However, long-term outcomes still remain a matter of discussion within the orthopaedic community. The purpose of this longitudinal prospective study was to evaluate radiographic and functional outcomes in patients with mild-to-severe HV who underwent Reverdin-Isham and Akin percutaneous osteotomy, following exostosectomy and lateral release.

**Methods:**

Eighty patients with mild-to-severe symptomatic HV were treated by MIS. Clinical evaluation was assessed preoperatively, as well as at 3 and 12 months after surgery and at final follow-up of 48 months, using the American Orthopaedic Foot and Ankle Society (AOFAS) hallux grading system. Patient satisfaction and complications were recorded. Computer-assisted measurement of antero-posterior radiographs was taken preoperatively, as well as at 3 and 12 months after surgery and at 48-month follow-up, analysing the intermetatarsal angle (IMA), the hallux valgus angle (HVA), the distal metatarsal articular angle (DMAA) and the tibial sesamoid position. Also, the bridging bone/callus formation was evaluated at the different radiographic follow-ups, while the articular surface congruency and the metatarsal index were calculated only preoperatively and at the last follow-up. Patient satisfaction was assessed using the visual analogue score (VAS). Statistical analysis was carried out using the paired *t* test. Statistical significance was set at *p* < 0.05.

**Results:**

The mean AOFAS score was 87.15 points at the final follow-up of 48 months, and the VAS score was 8.35/10. The post-operative radiographic assessments showed a statistically significant improvement compared with preoperative values. The mean corrections of each angular value at the last follow-up were as follows: IMA 3.90°, HVA 12.50°, DMAA 4.72° and a tibial sesamoid position of 1.10. The articular surface was congruent in 77 (96.25%) cases and incongruent only in 3 (3.75%). The complete healing of the osteotomies was achieved in all series at 3-month follow-up. However, the results obtained in the correction of the severe HV deformities were less encouraging.

**Conclusions:**

Minimally invasive surgery with Reverdin-Isham and Akin percutaneous osteotomy, in combination with previous exostosectomy and subsequent lateral soft-tissue release, is a safe, effective and reliable procedure for correction of mild-to-moderate HV. However, it requires a long learning curve because of the inherent difficulty of the mixed different surgical procedures.

**Trial registration:**

ClinicalTrials.gov PRS Protocol Registration and Results System: NCT02886221

## Background

Hallux valgus (HV) is a common, complex and progressive deformity of the forefoot with multiple clinical symptoms and a multifactorial aetiology [1]. Painful HV is more frequent in women between 40 and 60 years old, although it can appear in younger people because of biomechanical influence, hind and midfoot pathologies or sports activities, which might cause an overload on the first ray [2, 3]. For its correction, a wide variety of bony procedures are described, associated or not with soft tissue release, including osteotomies at the level of the head, midshaft and base of the first metatarsal, as well as arthrodesis of the first metatarso-cuneiform joint [4–7]. This demonstrates the complexity of the disease and the lack of a unique and most appropriate treatment, the choice of which continues to generate controversy [8].

At present, minimally invasive surgery (MIS) performed with minimal skin incisions (1–3 mm), an intraoperative image intensificator and without internal fixation [9] represents one of the most innovative approaches in forefoot surgery. This percutaneous dynamic management combines different procedures, most arising from the traditional open distal metatarsal osteotomy, in a mixed surgical strategy, according to the complexity of the deformity to be corrected [10–14]. These methods are rapidly becoming popular, as they are quick to perform, allow 1-day hospitalization, decrease post-operative morbidity as well as recovery and rehabilitation times, and chiefly because they are better accepted by patients [9, 15].

Although the most commonly performed percutaneous procedures have already been well described, providing equal effectiveness, sometimes superior, to traditional open surgery [16], their use is not equally accepted and their outcomes still remain a matter of discussion in the orthopaedic community, particularly in cases where no internal fixation is used [17, 18]. The Reverdin-Isham percutaneous osteotomy was described as a novel intra-articular medial closing wedge osteotomy of the distal metatarsal, in combination with an Akin osteotomy, both performed without fixation, to align the first ray by medial rotation of the first metatarsal head and distal metatarsal articular angle (DMAA) correction [15, 19–26]. Reverdin-Isham is not a complete osteotomy, as the MTT-1 lateral cortex is preserved; the closing wedge ensures contact of the metatarsal head with the metaphysis, and a special bandage is applied after surgery. In this way, no internal fixation is necessary. This allows the osteotomy to heal with the toe in its proper position, due to early weight bearing.

Since the end of the last century, MIS became widespread first in Spain and then in Europe by M. De Prado and P.L. Ripoll through their surgical practices and international theoretical-cadaveric courses, supported by the anatomical studies of Pau Golanó [20]. In 2002, the group GRECMIP (Groupe de Recherche et d'Enseignement en Chirurgie Mini-Invasive du Pied) began a project to develop and promote this new surgical treatment [26]. However, to the best of our knowledge, no previous study has evaluated the long-term results of this technique. Thus, the aim of this prospective study was to evaluate the radiographic and clinical outcomes of patients with mild-to-severe HV treated by MIS with Reverdin-Isham and Akin percutaneous osteotomy, following exostosectomy and lateral soft-tissue release.

## Methods

Between May 2010 and May 2012, a consecutive series of 80 Caucasian patients with diagnosis of mild-to-severe HV were enrolled in this prospective study at our institution and underwent the Reverdin-Isham percutaneous osteotomy, following percutaneous Akin osteotomy and percutaneous lateral soft-tissue release. All of these operative procedures were performed by a single surgeon, the senior author (C.B.), who followed and checked the patients personally during the post-operative period. All subjects participating in this prospective study received a thorough explanation of the risks and benefits of inclusion and gave their oral and written informed consent to publish the data. Approval from the General Clinical Directorate of our institution was obtained to introduce the novel technique before starting the operations. The study was performed in accordance with the ethical standards of the 1964 Declaration of Helsinki as revised in 2000 and those of Good Clinical Practice.

### Inclusion and exclusion criteria

Patients with diagnosis of mild to severe HV were enrolled consecutively and prospectively with precise inclusion criteria over a 2-year period. Ages ranged from 25 to 80 years. Only symptomatic patients with severe pain were included in this study. Exclusion criteria were as follows: congenital deformities of the foot, hallux rigidus, previous first ray trauma or foot and ankle surgery, diagnosis of rheumatic, dismetabolic, neurologic, infective or psychiatric pathologies. Furthermore, patients were excluded if they had painful fixed lesser toe deformities, signs of metatarsalgia or Morton’s neuroma.

### Surgical procedures

The different procedures for MIS HV correction, as adopted by our institution, were performed as described by De Prado [20]. Among these specific tools, various burrs of different size and form, adapted for Mm960 (produced by Medic Micro, Switzerland), a modular power driver for MIS, were used. During the operation, the patient was in a supine position, with the operated foot protruding from the table. No ankle joint tourniquet was applied, as it is not required for this technique. Prophylactic antibiotic (Cefazolin 2 g) was administered before surgery, and thromboembolic prophylaxis with Nadroparin Calcium injections was prescribed the same evening and for a 30-day period. Anaesthesia consisted in a conscious sedation in association with a regional ankle block, which combines five nerves: three superficial: saphenous, sural and superficial peroneal nerves, and two deep: tibial and deep peroneal nerves.

### Exostosectomy

**Fig. 1:**
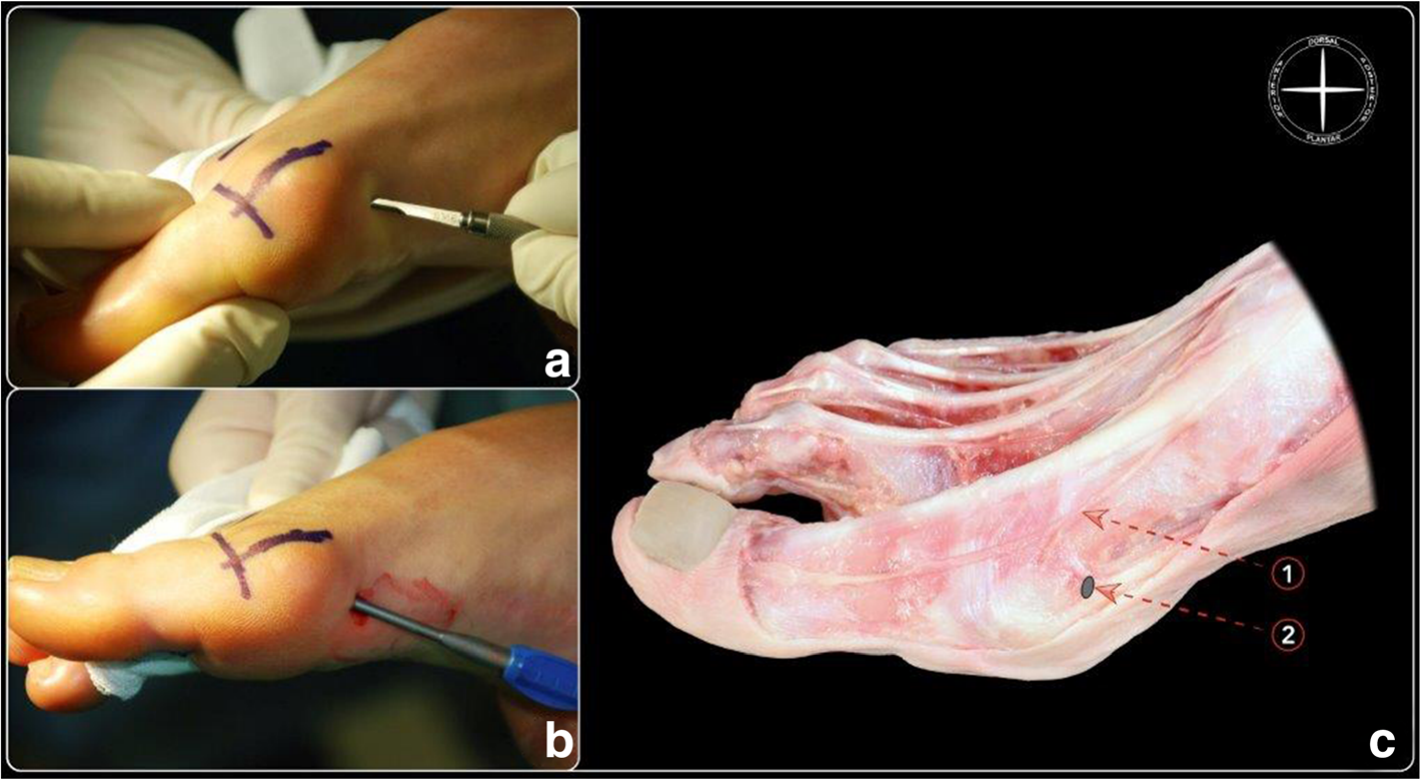
Portal placement (**a**) and rasp introduction (**b**). The protocolised incision protects the dorsomedial cutaneous nerve of the hallux (**c**): *1* dorsomedial cutaneous nerve of the hallux, *2* point of incision for Reverdin-Isham osteotomy

An incision of 3–5 mm long was made at the plantar side of the medial border of the first metatarsal head (Fig. 1a). Through this medial approach, a small scalpel was introduced within the joint capsule of the metatarso-phalangeal joint of the big toe. By a sweeping movement, the medial capsule was separated from the exostosis, subsequently using also a rasp (Fig. 1b). The location of this incision prevents damage of the dorsomedial cutaneous nerve of the hallux [20] (Fig. 1c). Then, a cylindrical burr (3.1 × 15 mm) was introduced, and the dorsal medial prominence was removed from the first metatarsal head until a flat surface was obtained, assessed under manual palpation and fluoroscopic control. Finally, the bony detritus was extruded manually.

### Reverdin-Isham osteotomy

**Fig. 2:**
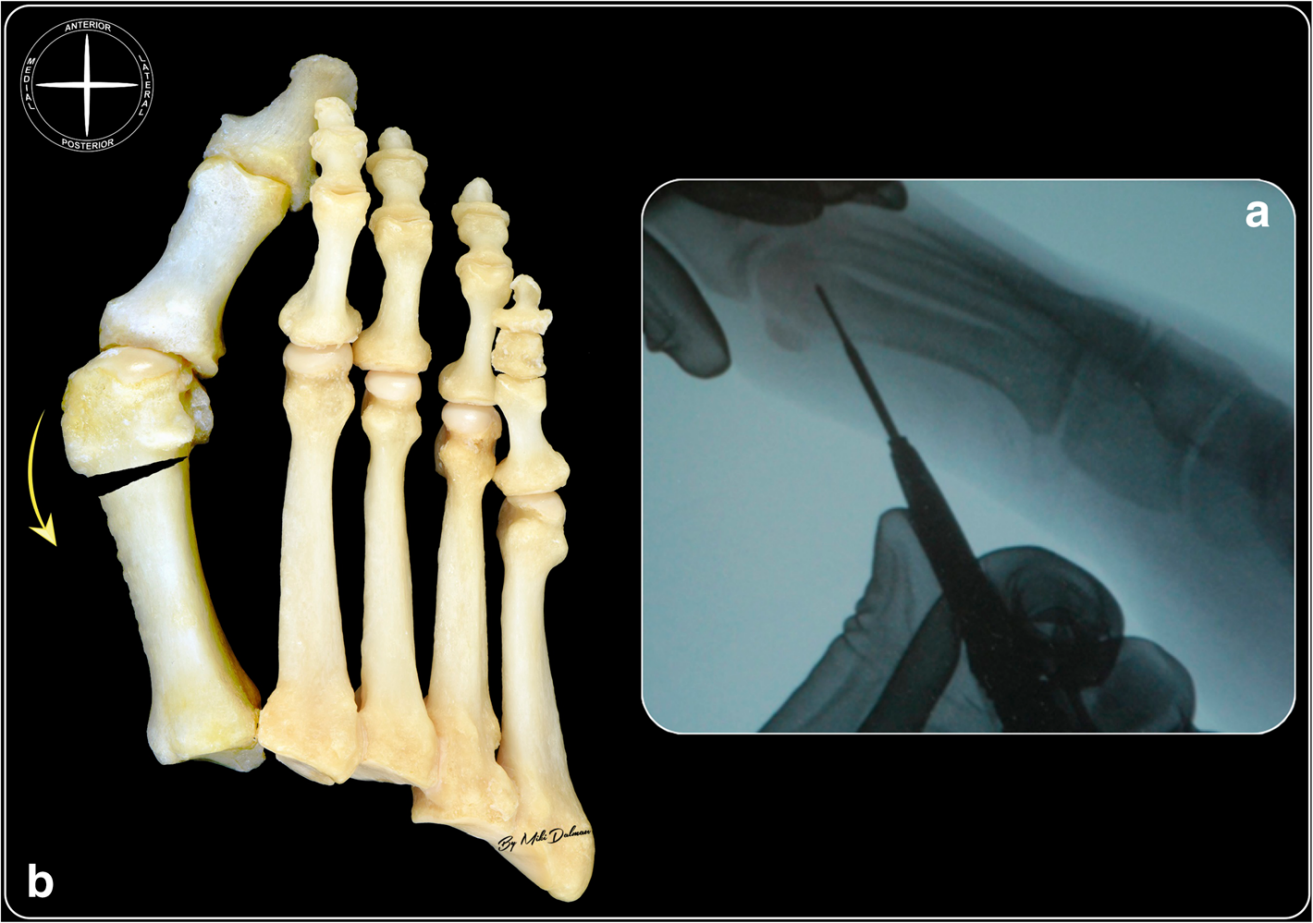
Reverdin-Isham osteotomy: intraoperative fluoroscopic imagine shoving the proper position and inclination of the burr to respect the distal first metatarsal bone (**a**). The final result of an ideal closing wedge osteotomy with a medial base that corrects also the DMAA (**b**)

Through the same incision used for the exostosectomy, a Shannon Isham burr (2 × 12 mm) was introduced at the junction of metaphysis and epiphysis. It was applied to the flat bone surface, achieved previously by exostosectomy, at an angle of approximately 45° to the long axis of the first metatarsal bone, keeping the articular cartilage surface of the first metatarsal head as reference point on the dorsal cortex, and the medial sesamoid bone as the reference point on the plantar cortex (Fig. 2a). In this position, under fluoroscopic control, the osteotomy was started following a distal-dorsal and proximal-plantar direction, extending until the lateral cortex, but without cutting it. At this point, the burr was slightly withdrawn in order to preserve a few millimetres of the lateral cortex, and the osteotomy of the plantar cortex was performed completely. Then, a Wedge burr (3.1 × 13 mm or 4.1 × 13 mm, depending on the DMAA value) was used to create a wedge with a medially oriented base. At the point of closing the wedge, osteoclasis of the preserved lateral cortex was achieved, modifying the orientation of the articular surface, normalizing the DMAA value and adding intrinsic stability to the osteotomy by producing contact of the trabecular bone (Fig. 2b).

### Tenotomy of the adductor hallucis tendon and lateral capsulotomy

A longitudinal skin incision was performed on the first web space, 2–3 mm lateral to the extensor hallucis longus tendon. The blade was longitudinally introduced in contact with the lateral surface of the base of the proximal phalanx; then, the blade was rotated 90° laterally and the first toe forced in varus, causing the adductor hallucis tendon to be sectioned and the lateral part of the capsule joint to be cut. Movement of the blade was carefully controlled in order to avoid a complete capsulotomy, which could produce joint instability.

### Akin osteotomy

Once lateral soft-tissue release was performed, a new incision 3 to 5 mm long on the lateral surface of the base of the proximal phalanx of the first toe was performed, just medial to the extensor tendons. Using a small scraper, the periosteum was removed from the lateral surface of the base of the proximal phalanx. Then, using a Wedge burr (3.1 × 13 mm), a wedge osteotomy (with medial base) was performed; as in the osteotomy on the head of the first metatarsal, the lateral cortex was preserved. Closing of the osteotomy and osteoclasis of the lateral cortex was achieved by means of a forced varus movement of the toe.

### Bandage

**Fig. 3:**
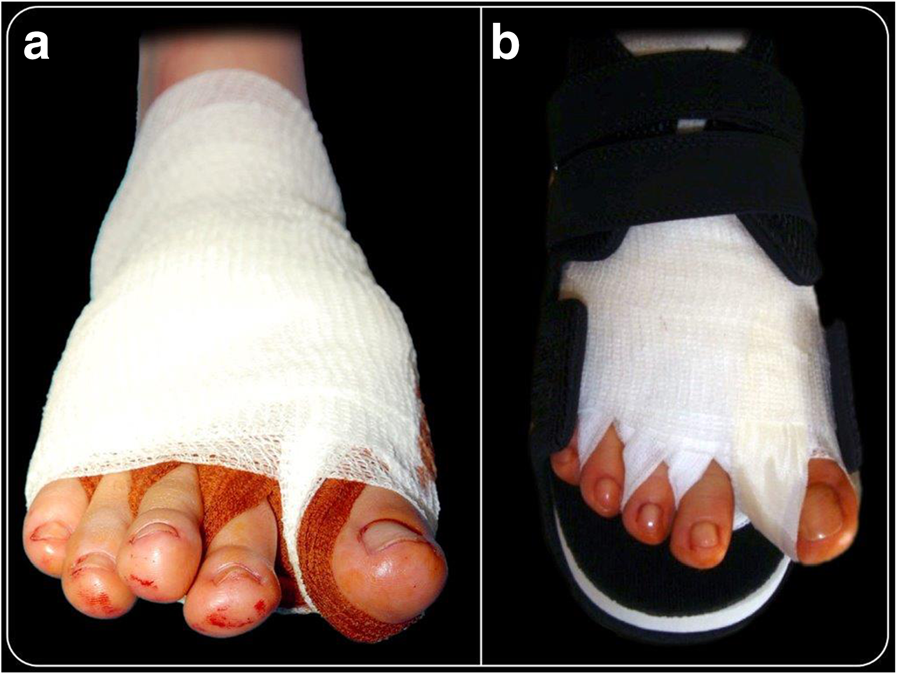
Example of post-operative bandage (**a**) and rigid flat-soled orthopaedic shoe (**b**)

After completing the surgery with suture of the capsule and cutaneous sutures of related cuts, a bandage was applied. Because there is no osteosynthesis material in this surgery, the bandage is a very important tool in order to maintain the correction obtained with the operation. Consequently, its application was performed with the utmost care and attention. The first toe was gently placed in overcorrection. Then, with a tape for bandages, the bend of the crisscross bandage was traced between the first and second toes, crossing them over the medial aspect of the exostosectomy in order to reinforce the strength of the bandage. Gentle traction was used to maintain the toe in light hypercorrection and plantar inclination. Finally, the forefoot was covered with tubular gauzes, except for the distal part of the toes and nails (Fig. 3a).

### Post-operative protocol

All patients followed the same post-operative protocol and were followed in the same standardized manner by the senior author (C.B.). The patients were allowed to walk as much as they could tolerate the same evening after surgery at discharge, using a rigid flat-soled orthopaedic shoe for the following 30-day period (Fig. 3b). Antero-posterior and lateral X-rays of non-weight-bearing feet were taken before the patients were discharged. We recommended a thromboembolic prophylaxis (Natrium Enoxaparin: 4000 IU/day) and an anti-edemigen therapy (Leucoselect, Lymphaselect and Bromelina: 1 cp/day) for 30 days, starting from the day of the surgery. Moreover, an analgesic therapy was prescribed for 2 weeks with Etoricoxib (90 mg, 1 cp/day) in the morning, also to prevent articular ossification; if pain persisted, Paracetamol/phosphate Codeine (1 g, max ×3/day) was prescribed. All of the patients were seen once a week for a month in our out-patient clinic. The first visit was 8 days after surgery. The original bandage was removed and substituted by a simpler bandage, but always with a slight overcorrection. During the three weekly visits, the bandage was changed in the same way. One month after surgical treatment, the bandage was totally removed, and after taking antero-posterior weight bearing and lateral X-rays (and sesamoid view when possible), an interdigital silicone orthoses space maintainer was positioned between the first and second toes. Patients were instructed to wear it for 3 months to help the first toe maintain its correct position until complete osteotomy consolidation. They were then able to walk with comfortable shoes, allowing total load on the operated foot. The only recommendations for the patient were to be careful with rough surfaces, sports and any other activities with forefoot overload. No specific physiokinesis therapy was suggested to restart daily activities.

### Patient assessment

The clinical and radiological analyses were carried out, respectively, by two independent investigators, the junior authors (M.F. and M.G.R.), not involved in the primary surgical treatment of the patients. The first is a resident of our clinic; the second is an orthopaedic surgeon of a different unit. For this study, all of the patients underwent clinical and radiographic assessment with the same protocol before surgery, as well as at 3 and 12 months and finally at 48 months after surgery, according to the American Orthopaedic Foot and Ankle Society (AOFAS) accepted guidelines [27]. For methodological reasons, the immediate post-operative X-rays at discharge, as well as the 1-month radiographic control, were not included for the radiographic evaluation: first, because it was a non-weight-bearing radiograph and, second, because, although it was weight bearing, in some cases, the sesamoid projection was not performed as the patients had pain or were afraid to excessively dorsiflex the big toe.

### Functional outcome measures

The clinical preoperative evaluation included a complete clinical history of the patients, their main characteristics (gender, age at the time of surgery, affected side) and physical examination of the foot. The 100-point AOFAS hallux-metatarso-phalangeal-interphalangeal scale [28] was used to assess clinical outcomes, and the difference (Δ) between preoperative and post-operative median values was calculated. Furthermore, all patients were investigated with the visual analogue scale (VAS). Additionally, any complications were recorded.

### Radiographic outcome measures

Routine standing antero-posterior, lateral and sesamoid X-ray views were obtained before surgery and at different follow-ups, according to our protocol. They were analysed at our institution in a standardised manner using electronically computer-assisted measurements for weight-bearing radiographs. The following parameters were evaluated: intermetatarsal angle (IMA: normal value <10°), proximal articular surface angle (DMAA: normal value <6°), hallux valgus angle (HVA: normal value <15°), tibial sesamoid position (using the recommended classification system by the American Foot and Ankle Society [29]), articular surface congruency, metatarsal index [30–32], callus formation in antero-posterior and lateral view radiographs and absence of radiolucent lines to determine bone union.

The relationship among the IMA, HVA values and tibial sesamoid displacement was then used to classify the deformities into three groups according to the presence of one of these Mann and Coughlin parameters [1, 28, 29, 33, 34]:

- Mild HV was defined as an IMA ≤11° and HVA <20° and less than 50% subluxation of the medial sesamoid (grade 1).
- Moderate HV was an IMA >11 but <16° and HVA of 20° to 40°, with 50 to 75% subluxation of tibial sesamoid (grade 2).
- Severe HV was an IMA ≥16° and HVA of >40° and more than 75% subluxation of tibial sesamoid (grade 3).

For each of these angles and tibial sesamoid positions, the difference (Δ) between preoperative and post-operative median values and the effectiveness of procedure correction (%) was calculated.

### Statistical analysis

Statistical analyses were performed by an independent statistician from the Department of Statistics at the University of Padua. The data is presented as the mean (plus standard deviation) or median (range) for continuous variables and as numbers for categorical measures. For the statistical evaluation of the angular values and the clinical scores obtained with the AOFAS scale pre-intervention and different follow-ups, we used the Student *t* test. For angular values not normally distributed, we used the Wilcoxon test of signed ranks. The change in position of the medial sesamoid was analysed by testing the symmetry of Bowker, an extension of the McNemar test for tables larger than 2 × 2. All *p* values were two-sided, using a significance level of *p* < 0.05.

## Results

Eighty feet, 43 right and 37 left, of 80 consecutively enrolled patients, met the inclusion criteria and were considered in the analyses. The median patient age at the time of the surgery was 51 ± 15.5 years (range 26–78). There were 75 women (93.4%) and 5 men (6.6%). None of the patients was lost during the different follow-ups.

### Clinical outcomes

At the preoperative evaluation, the mean total AOFAS score of the patients treated was 54.1 ± 8.3 points (range 39–85). The median of the results was 52 points, and only 12 cases obtained over 60 points. Limitation in daily and recreational activities was implicated in 74 cases (92.50%).

**Fig. 4:**
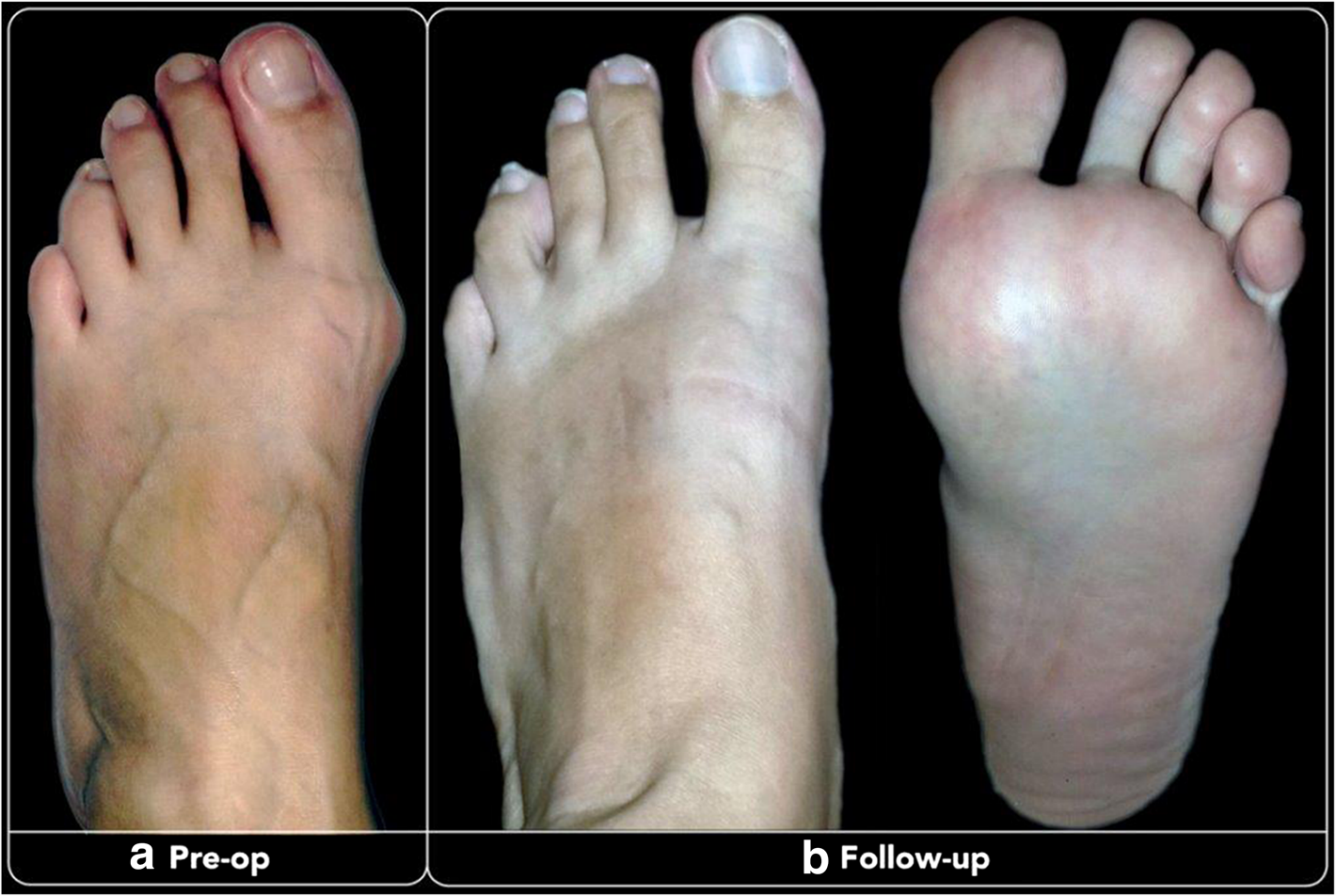
Pre-op (**a**) and at 48-month last follow-up (**b**) clinical images of a 36-year-old woman after having undergone percutaneous Reverdin-Isham osteotomy, lateral release and Akin osteotomy for mild HV correction

**Fig. 5:**
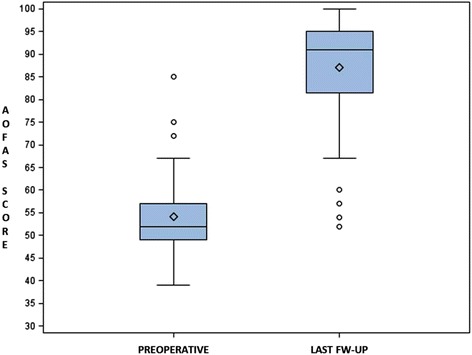
Graph of statistical analysis of preoperative and post-operative AOFAS scores (*P* ≤ 0.05)

At the different follow-ups until the final one, the mean total AOFAS score of the patients treated improved progressively and significantly (Tables 1 and 2; Figs. 4 and 5): 72.20 points (range 44–100) at 3-month follow-up, 78.60 points (range 44–100) at 12-month follow-up and 87.15 points ± 12.83 (range 52–100) at the final follow-up (*p*< 0.0001). At the final follow-up, the pain was mild, occasional or absent in 73 cases (91.25%). Only two patients (2.50%) still had difficulty or limitation in daily and recreational activities. At the final follow-up period, the mean VAS score was 8.35/10 (3–10).

**Table 1:** AOFAS score at different follow-ups

	Preoperative	3 months	12 months	48 months	*p* value
Mean AOFAS score (pts)	54.1 (±8.3)	72.2	78.6	87.1 (±12.8)	*p* < 0.0001

**Table 2:** AOFAS score before surgery and at final follow-up (48 months)

	Preoperative (%)	Last follow-up (%)
Pain		
None	2.5	62.5
Mild, occasional	19	28.75
Moderate, daily	78.5	8.75
Severe, almost always present	0	0
Activity limitations		
No limitations	7.5	76.5
Limited daily and recreational activities	60	21
Severe limitation	32.5	2.5
Footwear requirements		
Fashionable, conventional shoes	13.5	44
Comfort footwear, shoe insert	82.5	56
Modified shoes or brace	4	0
MTP joint motion		
Normal or mild restriction >75°	82.5	61
Moderate restriction 30°–74°	17.5	37.5
Severe restriction <30°	0	1.5
Callus related to hallux MTP-IP		
No callus or asymptomatic callus	39	93.75
Callus symptomatic	61	6.25
Alignment		
Good, hallux well aligned	0	68.75
Fair, no symptoms	0	23.7
Poor obvious symptomatic malalignment	100	7.5

### Radiographic outcomes

**Fig. 6:**
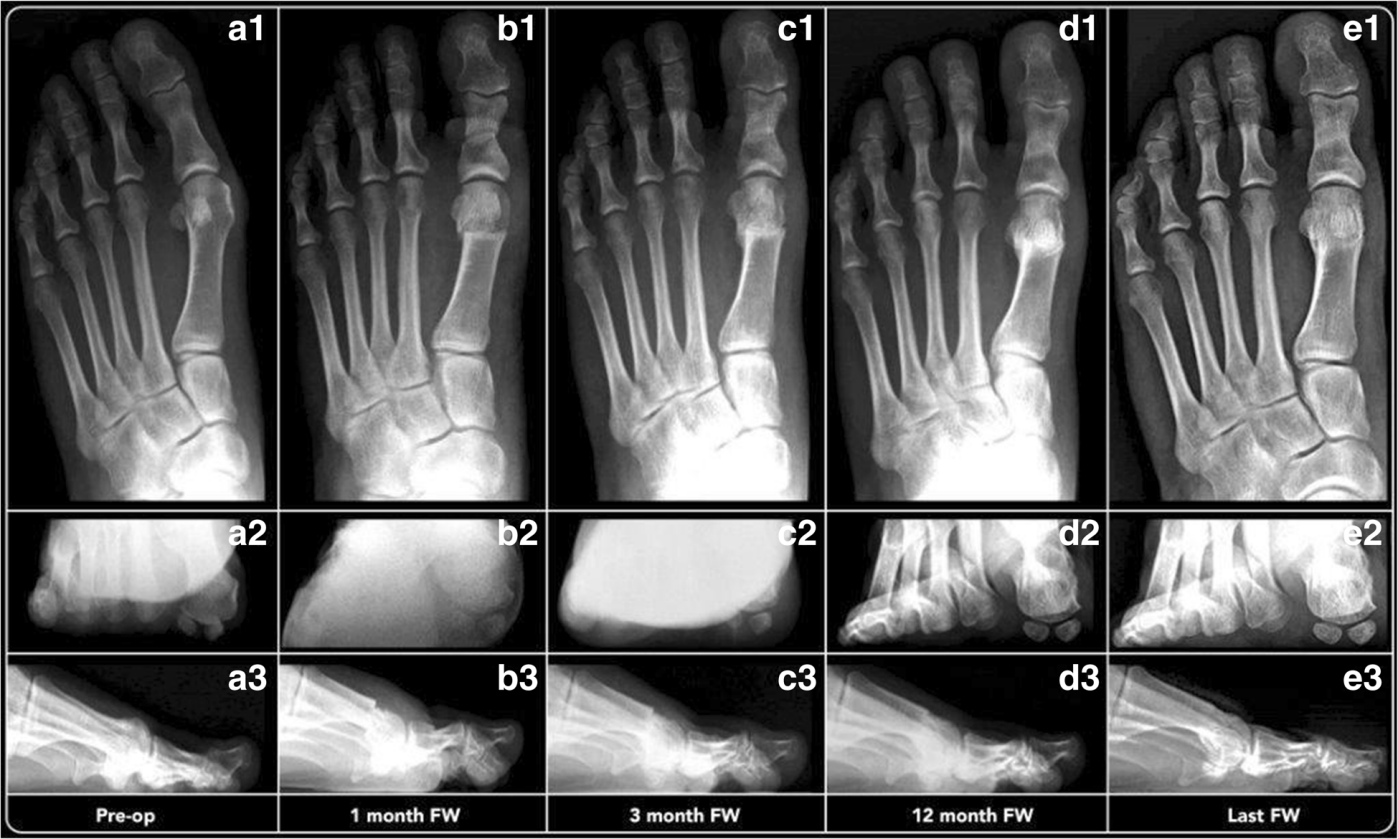
A 36-year-old woman with mild HV: *1* antero-posterior radiographic images, *2* sesamoid and *3* lateral view at preoperative period (*a*), 1-month follow-up (*b*), 3-month follow-up (*c*), 12-month follow-up (*d*) and 48-month follow-up (*e*), showing the maintained correction of the deformity

According to the Mann and Coughlin grading system, 7 (8.75%) patients were classified in group A (mild HV), 56 (70.0%) in group B (moderate HV) and 17 (21.25%) in group C (severe HV). The radiographic outcomes of our cohort are summarized in Table 3, while the radiographic results for each subgroup are reported in Table 4. Regarding bone/callus formation, complete healing of the osteotomies was noted in all series at 3-month follow-up (Fig. 6).

**Table 3:** Angular values (IMA, HVA and DMAA), sesamoid position, metatarsal index and congruency of the metatarso-phalangeal-I articular surface

	Pre-op	3-month FU	12-month FU	48-month FU	Efficacy (%)
IMA (degrees)					
Tot. (DS)	*12.9* (*2.8*)	*9.0*	*9.0*	*9.0* (*2.0*)	*30.23*
Mild	9.1			7.1	22.0
Moderate	12.1			9.0	27.3
Severe	16.6			10.0	39.2
HVA (degrees)					
Tot.	*26.4* (*6.7*)	*12.3*	*13.1*	*13.9* (*6.2*)	*47.35*
Mild	16.4			9.8	40.2
Moderate	26.0			14.2	45.0
Severe	32.0			14.7	54.0
DMAA (degrees)					
Tot.	*10.12* (*4.3*)	*5.0*	*5.2*	*5.4* (*3.2*)	*46.64*
Mild	6.3			3.9	38.1
Moderate	10.0			5.3	47.0
Severe	12.4			6.5	47.6
Sesamoid Position (pts)					
Tot.	*2.4* (*0.6*)	*1.1*	*1.2*	*1.3* (*0.6*)	*45.83*
Mild	2.0			0.9	55.0
Moderate	2.3			1.3	43.5
Severe	2.8			1.7	39.3
Metatarsal Index					
M1 < M2	34			58	
M1 = M2	28			19	
M1 > M2	18			3	
MTP-I Art. Sup.					
Congruent	61			77	
Incongruous	19			3

**Table 4:** Preoperative and last follow-up (48 months) angular values

	Mild HV	Moderate HV	Severe HV	*p* value
IMA				
Preoperative	9.1 (±0.7)	12.1 (±1.9)	16.6 (±2.1)	*p* < 0.0001
Last follow-up	7.1 (±0.8)	9.0 (±1.9)	10.0 (±2.2)	*p* < 0.0001
HVA				
Preoperative	16.4 (±3.1)	26.0 (±4.93)	32.0 (±7.7)	*p* < 0.0001
Last follow-up	9.8 (±5.8)	14.2 (±5.9)	14.7 (±7.3)	*p* < 0.0001
DMAA				
Preoperative	6.3 (±1.22)	10.0 (±4.0)	12.4 (±4.7)	*p* < 0.0001
Last follow-up	3.9 (±2.5)	5.3 (±3.0)	6.5 (±3.9)	*p* < 0.0001
Sesamoid position				
Preoperative	2.0 (±0.6)	2.3 (±0.6)	2.8 (±0.4)	*p* < 0.0001
Last follow-up	0.9 (±0.7)	1.3 (±0.6)	1.7 (±0.7)	*p* < 0.0001

#### Intermetatarsal angle (IMA)

**Fig. 7:**
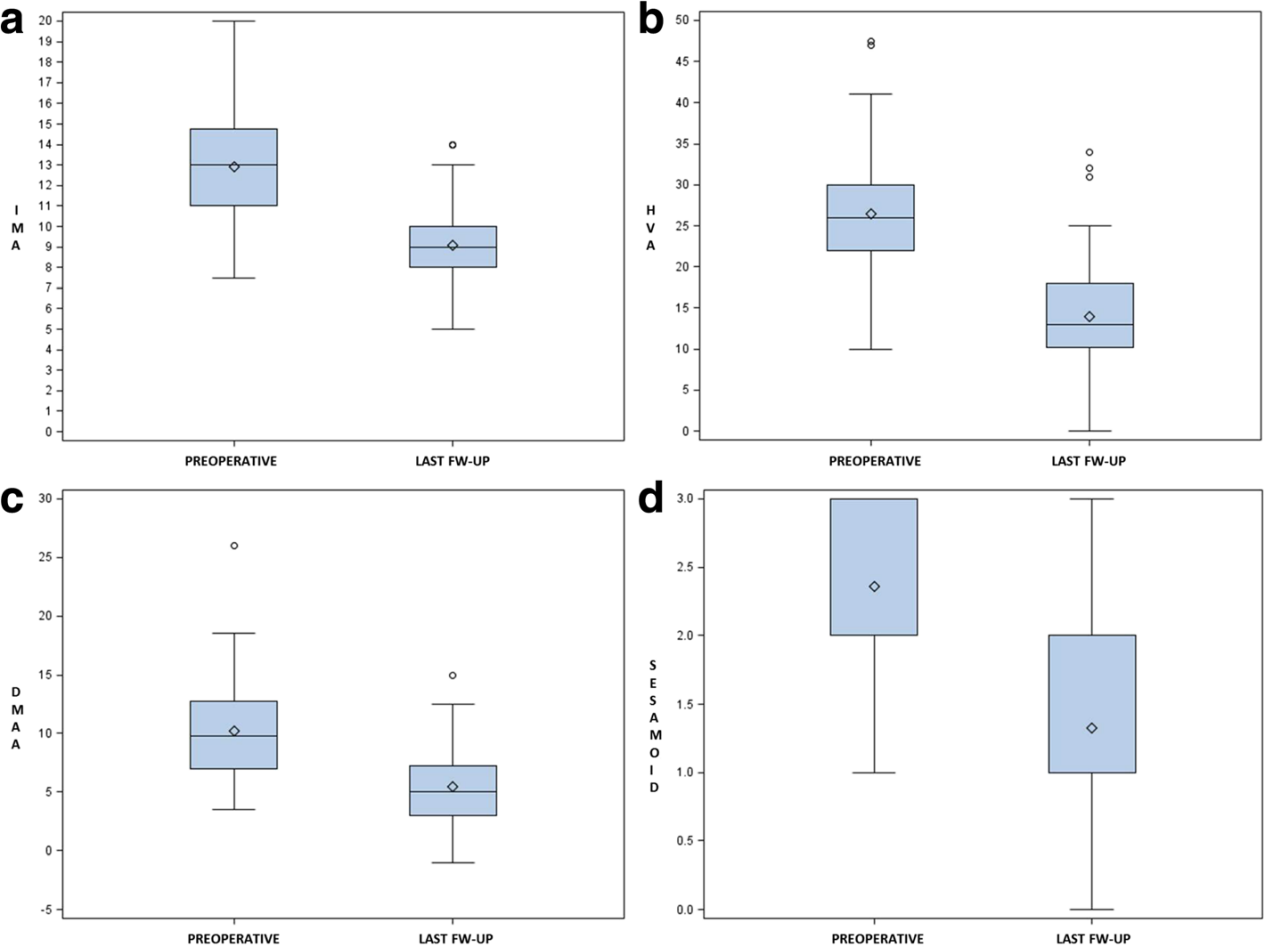
Graph of statistical analysis of preoperative and post-operative different angular values (*p* ≤ 0.05): IMA (**a**), HVA (**b**), DMAA (**c**) and tibial sesamoid position (**d**)

The mean IMA value decreased from 12.90° ± 2.83° (range 7.50°–20.00°) preoperatively to 9.00° ± 2.04° (range 5°–14°) at the 48-month follow-up (Fig. 7a), with a mean correction of 3.90° and an effectiveness of 30.23% (*p* < 0.05).

#### Hallux valgus angle (HVA)

The mean preoperative HVA was 26.40° ± 6.75° (range 10°–47.5°). The mean value at the 48-month follow-up assessment was 13.90° ± 6.25° (range 0°–34.00°), with a mean correction of 12.50° (Fig. 7b) and an effectiveness of 47.35% (*p* < 0.05).

#### Distal metatarsal articular angle (DMAA)

The mean preoperative DMAA was 10.12° ± 4.26° (range 3.5°–26.00°). The mean value at the 48-month follow-up examination was 5.40° ± 3.19° (range −1.00° to 15.00°) with a mean correction of 4.72° (Fig. 7c) and an effectiveness of 46.64% (*p* < 0.05).

#### Medial sesamoid position

The mean preoperative dislocation of the medial sesamoid was 2.40 ± 0.64 (range 1–3). Its mean value at the 48-month follow-up assessment was 1.30 ± 0.63 (range 0–3), with a mean correction of 1.10 (range 0–3) and an effectiveness of 45.83% (*p* < 0.0001) (Fig. 7d).

#### Articular surface congruency

In the preoperative period, 61 patients (76.25%) had a congruent articular surface and 19 (23.75%) incongruent. At final follow-up, 77 patients (96.25%) had a congruent articular surface and only 3 (3.75%) incongruent.

#### Metatarsal index

In the preoperative period, the metatarsal index of patients was Minus (M1 < M2) in 34 cases (42.5%), Plus Minus (M1 = M2) in 28 cases (35.0%), and Major (M1 > M2) in 18 cases (22.5%). At the last follow-up, it resulted Minus (M1 < M2) in 58 cases (72.5%), Plus Minus (M1 = M2) in 19 cases (23.75%), and Major (M1 > M2) in 3 cases (3.75%).

### Complications

Complications occurred in 25 patients (31.25%): six major and 19 minor. The major complications (7.5%) included five cases of recurrence and one case of severe stiffness (ROM <30°). The minor (23.75%) complications were slight loss of normal range of MTP joint motion (ROM 30°–74°) in 16 cases. In three other cases, minor complications were resolved over time. Because of portal burns during operation, two patients presented delayed wound healing, which healed completely in 4 weeks and did not required subsequent surgery. One patient complained of dysesthesia of the skin distal to the interphalangeal joint of the big toe because of neuritis of a cutaneous sensory dorsal branch, an infrequent complication caused by incorrect surgical access, which had resolved spontaneously by the final follow-up. We did not encounter any cases of hallux varus due to overcorrection, malunion, delayed union or non-union. There were no cases of thrombo-embolism, no cutaneous or deep infections nor avascular necrosis of the metatarsal head. No case of dorsal displacement of the metatarsal head was recorded in this study.

## Discussion

Although in the last few years, the number of studies regarding the effectiveness of MIS in HV correction has increased [35–37], to the best of our knowledge, this is the first prospective, single-centre study reporting outcomes of Reverdin-Isham percutaneous osteotomy in a consecutive, single surgeon, large patient series with a median follow-up of 48 months. Furthermore, no other report in the literature has assessed the results of this procedure at three different follow-ups. The study was designed to evaluate, on the basis of clinical and computer-assisted radiographic data, the validity and reliability of this percutaneous techniques for correction of mild to severe HV deformity.

In our cohort, the mean AOFAS score improved significantly until the last follow-up (Tables 1 and 2). An increase of 18.1 points 3 months after surgery was seen, further 6.7 points at 12 months and 33 points at the last follow-up. Moreover, more than half of the patients scored more than 90 points, while the median, statistically the more “powerful indicator,” was 91 points, in contrast to 52 points in the preoperative period. This clinical improvement was also evident in all patient sub-groups, almost in the same way, maintaining unaltered the gradient correlated to the degree of the deformity. In the sub-groups, the mean AOFAS score at the last follow-up was 87.1 points (Δ = +32.1) in the mild HV group, 86.6 (Δ = +32.7) in the moderate HV group and 83.7 (Δ = +30.2) in the severe one. Overall, the AOFAS score found in our study was comparable to those reported by different authors not only using minimally invasive techniques [10, 12, 13, 25, 26, 38], with or without osteosynthesis, but also with series of open surgical procedures using Chevron, Scarf or proximal metatarsal osteotomies [10, 13, 19, 39, 40]. In particular, our data was similar for patient demographic aspects and complications, including stiffness; however, our group of patients had a larger sample size and follow-up duration [10, 13, 19, 39]. At the last follow-up, only seven patients reported daily pain, compared to 63 cases (78.5%) of the preoperative period, while most of the subjects (73 cases; 91.5%) reported no pain or only mild occasional pain (Table 2). These percentages are similar to those of other studies [10, 24, 25]. Despite the anti-edema prophylaxis adopted, 40% of our subjects complained of swelling of the foot and ankle, which remained for more than a month after the operation. Apparently, the causes of both prolonged pain and swelling in these cases were related to the delayed formation of bone callus until the complete healing of the osteotomies seen at 3-month follow-up.

According to the AOFAS scale, the alignment was considered good in 55 cases (69%), discrete in 19 cases (23.5%) and poor in 6 (7.5%). Overall, in 92.5% of our cases, there was improvement, which is comparable with that reported in the literature [10, 25, 35, 38–44] (Figs. 5 and 6).

One of the possible side effects of Reverdin-Isham percutaneous osteotomy, as it is an intra-articular medial closing wedge osteotomy, is the stiffness of the first metatarso-phalangeal joint [45, 46], which was noticed also in our cohort. Before the operation, 66 cases (82.5%) presented with preserved range of motion and the other 14 cases (17.5%) with slight limitation. After surgery, the joint movement was completely normal in 49 cases (61%) and was slightly reduced in another 30 cases (37.5%), while severe limitation to the flexion-extension was present only in an elderly patient. In accordance with Bauer et al., the potential cause could be the remains of bony fragments in the joint and the capsular tissues, produced during the extensive exostosectomy and the lack of accurate cleaning of the work area with rasps and irrigation with normal saline solution [35].

There were three intraoperative complications, which had resolved spontaneously by the final follow-up: one neuritis of a cutaneous sensory dorsal branch and two skin burns around the portal. Several studies have been reported in the literature [47, 48] comparing the complication rate between diathermy and scalpel for skin incision, without showing any significant differences. However, to the best of our knowledge, no study has examined the complication rate of surgical burr in MIS. In our experience, it is a fundamental surgical aspect to avoid putting too much manual pressure on the burr during the performance of osteotomies, rather accompanying it gently with the fingers during the entire process; otherwise, the resistant bone can push the burr to the edges of the portal, causing skin burn.

With regard to radiographic analysis (Table 3), all correction angular values obtained in our cohort were statistically significant (*p* < 0.0001). Although the distal closing wedge osteotomy has been described without resulting in any lateral translation of the metatarsal head, the IMA decreased from a mean value of 12.9° at preoperative examination to a mean value of 9.0° at the final follow-up, with a difference of 3.9° and an efficacy of 30.5% in angular value correction. In agreement with Bauer et al. [10, 21, 35], the Reverdin-Isham osteotomy has a slight impact on this angle, reporting an IMA mean improvement of about just 3°, again a better mean correction of about 8° and 15° for the DMAA and HVA, respectively. According to our experience, the efficacy in IMA correction is probably explained by the combined action between the three different surgical steps of the procedure. First, during the application of manual force to perform the lateral cortex osteoclasis at the step of the wedge closing, a minimum translation of the metatarsal head occurs (Fig. 6 B1–E1). It is known that distal osteotomies allow achieving proximally 1° of IMA correction for each millimetre of metatarsal head lateral translation [49, 50]. Second, the tenotomy of the adductor hallucis tendon and lateral capsulotomy contributes to the lateral movement of the first metatarsal axis, further decreasing its varus. Third, Isham in his original paper of 1991, stated that the average reduction of the IMA is especially noted when the procedure is performed in association with Akin osteotomy [19].

For the HVA, the effectiveness in angular value correction was 47.4%, with a preoperative mean value of 26.4° and 13.9° at the last follow-up, respectively. One of the objectives of distal osteotomies is to reduce the DMAA by a medial rotation of the metatarsal head. In the analysed sample, average correction efficacy was 46.1%; the mean preoperative angular value was 10.2° and 5.4° at the final follow-up. According to Coughlin [29], it is very important to correct DMAA, since a stable recovery can be achieved only by setting up the bone structure, re-orienting articular surfaces and re-equilibrating muscle forces of the first ray, avoiding the retraction of soft tissue and peri-articular adhesions. As reported in the literature [10, 24, 35, 39, 42], confirmation of the re-orientation of the forces on the muscle-ligamentous compartment was seen as restoration of the articular congruency and sesamoid compartment alignment.

Reverdin-Isham percutaneous osteotomy was a reliable procedure in correcting the different radiological parameters considered in this analysis, and our data are comparable with the reported angular corrections obtained with other percutaneous or open distal metatarsal osteotomies [13, 39, 42, 51]. Derotation of the metatarsal head (DMAA), and the anatomical reduction of the tibial sesamoid, necessary to prevent the recurrence of valgus [52], were all maintained until the last follow-up (Table 3). However, the results obtained in the correction of the HV severe deformities were less encouraging. In fact, the correction efficacy of the different single angles analysed, although high, was in some cases not sufficient to report them as in the normal range (Table 4). The six major complications of our series occurred in the severe forms, with an IMA greater than 15°. Hence, in accordance with Bauer et al. [10], we believe this is the angular value limit, beyond which the use of only Reverdin-Isham osteotomy as described is not recommended.

The first strength of this study is its nature: a prospective evaluation of a consistent group of 80 patients with the same fixed follow-up points. A 48-month follow-up can be considered a long follow-up period compared with previous published studies. Further, all operations and the post-operative controls were always performed in the same way by the same surgeon (C.B.). All clinical and radiographic data were collected and analysed separately by the same two independent investigators, who were not involved in the patients’ treatment and one not belonging to our unit. The main limitation of this study is the lack of a control group, which would be useful to compare the results of this percutaneous technique. However, as reported by several authors [15, 42, 44, 53], MIS includes different techniques, and the heterogeneity of the groups examined in various reports does not permit at present an effective comparison and clear conclusions. For these reasons, we believe that long-term follow-up with multicentre studies and randomized controlled clinical trials comparing Reverdin-Isham osteotomy outcomes to those of other percutaneous methods would provide useful information for the validity and reliability of MIS in the treatment of forefoot deformities.

## Conclusions

Based on our experience in MIS with the first cohort of patients described in this report, we conclude that Reverdin-Isham and Akin percutaneous osteotomy in combination with previous exostosectomy and following lateral soft-tissue release is a safe, effective and reliable procedure for correction of mild-to-moderate symptomatic HV. Good results remained consistent at the midterm follow-up point. The most important aspects that should encourage the use of this percutaneous technique are its minimally invasive nature, the low number of complications, the absence of osteosynthesis, distal ankle block anaesthetic technique, early weight bearing and good cosmetic results with minimal surgical scars.

## Abbreviation

AOFAS: American Orthopaedic Foot and Ankle Society
DMAA: Distal Metatarsal Articular Angle
GRECMIP: Groupe de Recherche et d'Enseignement en Chirurgie Mini-Invasive du Pied
HV: Hallux Valgus
HVA: Hallux Valgus Angle
IMA: Intermetatarsal Angle
MIS: Minimally Invasive Surgery
VAS: Visual Analogue Scale
